# The Burden of Rotavirus Gastroenteritis in Children: A Hospital-Based Prospective Study in Western Rajasthan

**DOI:** 10.7759/cureus.11020

**Published:** 2020-10-18

**Authors:** Suresh Kumar Meel, Vikash Katewa, Romil Singh, Alka Bishnoi, Pramod Sharma, Sawai Singh Rathore, Dhwani Kamrai, Kaushal Shah

**Affiliations:** 1 Pediatrics, Dr. S.N. Medical College, Jodhpur, IND; 2 Internal Medicine, Metropolitan Hospital, Jaipur, IND; 3 Internal Medicine, Dr. S.N. Medical College, Jodhpur, IND; 4 Psychiatry, Griffin Memorial Hospital, Norman, USA

**Keywords:** rotavirus, enzyme linked immunosorbent assay (elisa), gastroenteritis, feeding infants and young children

## Abstract

Objective

Rotaviruses are the prime cause of gastroenteritis amongst infants and young children worldwide. In India, the mortality and economic impact caused by rotavirus are high. The objective of this is to understand the burden of rotavirus in acute watery diarrhea and its circulating genotypes in hospitalized children less than five years of age for acute gastroenteritis in western Rajasthan.

Methodology

This is a hospital-based prospective study conducted in the pediatrics department of Dr. Sampurnanand (S.N.) Medical College of Jodhpur in India for one year during 2018. The study included 399 children less than five years old, presenting with acute gastroenteritis who needed to be admitted for at least six hours. We enrolled subjects after obtaining informed consent from the guardian. Stool samples of 5 gm or ml were collected in a sterile container and stored at minus 20 degrees centigrade while transporting to Christian Medical College (CMC) virology lab in Vellore, India. The stool samples were subjected to Enzyme-Linked Immunosorbent Assay (ELISA) testing, followed by genotype determination. We investigated data through statistical analysis from all collected data.

Results

A total of 399 patients fulfilled the enrollment criteria; out of them, 92 (23.05%) were positive for rotavirus, and maximum cases were seen in the age group of six months to two years (78.26%). Rotavirus positivity was more in males (64.13%) than females (35.86%). The rotavirus infection was seen throughout the year, with a peak in cases from November to February (73.91%). G3P8 (55.43%) was the most common strain causing rotavirus diarrhea, followed by G1P8 (9.72%) and G3+G12P8 (8.69%). Based on the Vesikari clinical severity score, 70.65% of patients had severe diarrhea.

Conclusion

This prospective study highlights the healthcare and economic burden of rotavirus, especially in children of less than five years. The incidence of rotavirus is observed in winter months, and its prevalence in all cases of acute diarrhea in our study is 23.05%. G3P8 was the most common genotype causing rotavirus diarrhea in our region in both non-vaccinated and vaccinated children, followed by G1P8 and G3+G12P8, respectively.

## Introduction

Rotavirus is the most common cause of acute gastroenteritis (AGE) and severe dehydration among children of less than five years of age in India [1,2]. About 11.37 million illnesses, 3.27 million outpatient visits, and 872,000 inpatient admissions occur each year due to rotavirus infection. India spends approximately 2.0 to 3.4 billion Indian Rupees, which is roughly 41 to 72 million United States dollars annually to treat rotavirus diarrhea in children <5 years of age [2]. Furthermore, an estimated 78,000 rotavirus-associated deaths occur among Indian children annually, according to 2011 estimates [3]. The rotavirus belongs to the Reoviridae family. Rotavirus is a non-enveloped double-stranded RNA genome, consisting of 11 gene segments within a multilayered protein capsid [1,3]. Out of 11 gene segments, six are structural proteins (VP1, VP2, VP3, VP4, VP6, and VP7) organized in a concentric layer around the genome, and the rest of the gene segments are non-structural proteins (NSP). The outermost layer of the virus particle is composed of VP7 and VP4 proteins, both of which induce neutralizing antibodies.

The most common circulating rotavirus genotype worldwide and Finland is G1P8, yet the genetic diversity within circulating rotavirus genotype G1P8 rotavirus strains is present with several intra-genotyping lineages and their sublineages. As a whole, the diversity of rotaviruses is generated by several mechanisms, including point mutations and gene rearrangements. Vaccination against rotavirus is very crucial since the gastroenteritis caused due to it is a threat to the healthcare system with a substantial financial burden on the developing economies. Several health organizations across the globe have promoted rotavirus vaccination. Guidelines and recommendations are provided by the World Health Organization (WHO), the European Society for Paediatric Gastroenterology, Hepatology, and Nutrition, and by the European Society for Paediatric Infectious Diseases [1,4]. As data is unavailable for the rotavirus positivity from western Rajasthan, the current study was planned to determine the burden of rotavirus in acute watery diarrhea and its circulating genotypes in hospitalized children of less than five years for acute gastroenteritis.

## Materials and methods

Study setting

It is a hospital-based prospective observational study conducted at the Umaid and MDM Hospitals in the Department of Pediatrics, Dr. S N Medical College, Jodhpur, over a period of one year from August 16, 2017, to August 15, 2018.

Inclusion criteria

The inclusion criteria are mentioned in Table 1.

**Table 1:** Inclusion criteria

Inclusion Criteria	
1	Presents to our hospital for treatment of Acute Gastroenteritis (AGE) and was treated with oral or intravenous fluids at the emergency department for at least 6 hours
2	Admitted to the hospital and treated with oral rehydration or intravenous fluids for AGE
3	Was <5 years of age
4	Was able to submit a stool sample (for testing) during the first 48 hours after presentation

Exclusion criteria

The exclusion criteria are mentioned in Table 2.

**Table 2:** Exclusion criteria

Exclusion Criteria	
1	Children who Were >5 years of age
2	Unable to contact parent/caregiver or guardian or unable to obtain informed consent
3	Chronic diarrhea/bloody diarrhea/dysentery
4	Admitted to another hospital for more than 24 hours and subsequently transferred to the current hospital
5	Not able to collect a stool sample

Acute Gastroenteritis (AGE) case definition 

The AGE is defined as the occurrence of ≥3 episodes of diarrhea (stools of a less formed character than usual) within 24 hours, less than seven days before the hospital visit, which is not explained by an underlying medical condition. 

Confirmation of rotavirus cases

Any case of AGE testing positive for rotavirus on stool testing by Enzyme-Linked Immunosorbent Assay (ELISA).

Clinical assessment

History of fever, loose stools, vomiting, and the duration of presenting complaints was obtained from the parent or guardian. Anthropometry was done to find out the grade of malnutrition. Children were assessed for dehydration signs and classified into severe, some, or no dehydration [1,4].

Stool collection and laboratory analysis 

Children of less than five years with acute gastroenteritis and who were admitted for at least six hours were enrolled for this study after informed consent, and 5 gm to 10 gm or ml stool samples were collected in a sterile container and stored at -20 degree centigrade before and during transport to virology lab at CMC, Vellore. The stool samples were subjected to ELISA testing, followed by genotype determination (PCR).

Statistical analysis

The data were analyzed with Microsoft Excel 2007 (Microsoft Corporation, Redmond, USA), and statistical analysis was performed with window Statistical Product and Service Solutions (SPSS) version 23 (IBM Corp, Armonk, USA). The categorical data findings were represented in percentage, and numeric data results in mean with standard deviation and median. Descriptive statistics were used to summaries data through frequency, mean, standard deviation, and percentages. A comparison of two groups was made with the help of Chi-square (x2) and t-tests. The p-value was determined and considered statistically significant if it is below 0.05.

## Results

**Figure 1:**
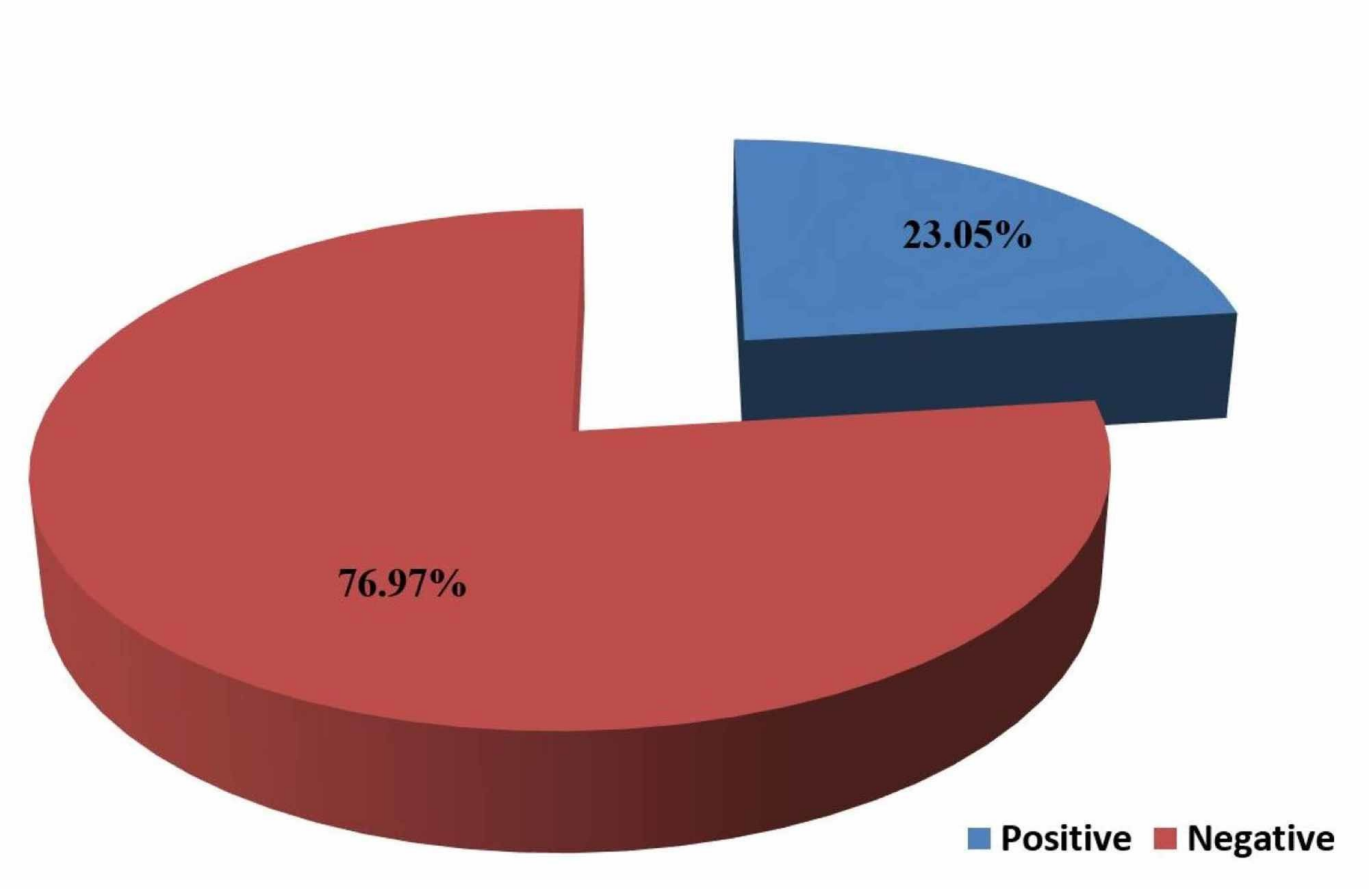
Prevalence of rotavirus diarrhea in the study group

A total of 399 children were included, out of which 92 (23.05%) were positive for rotavirus by ELISA test, as seen in Figure 1.

**Figure 2:**
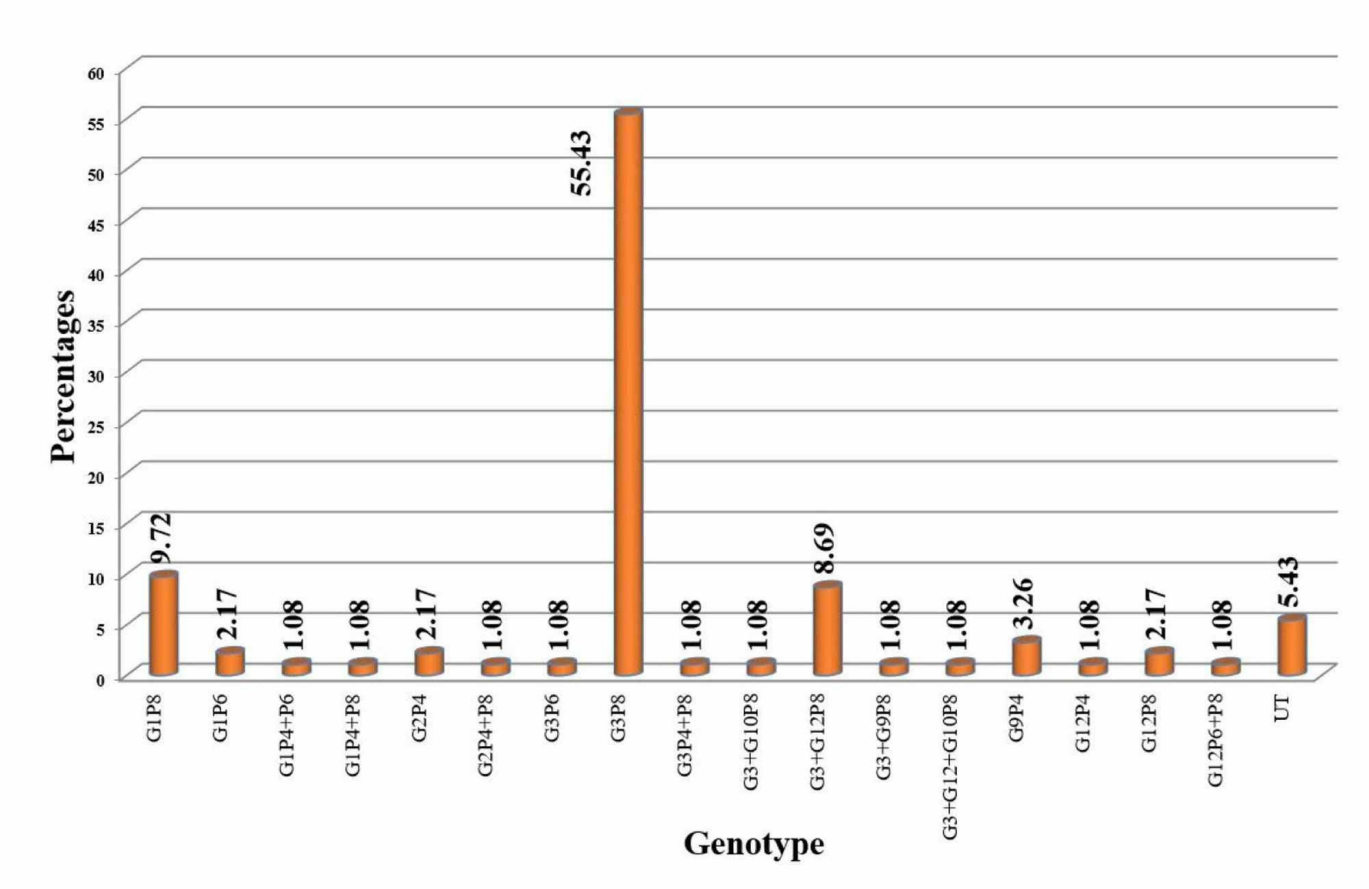
Distribution of case according to genotyping

In this study, the results showed that G3P8 was the predominant strain (55.43%), followed by G1P8 (9.72%) and G3+G12P8 (8.69%). About 5.43% of cases of rotavirus diarrhea were untypable, as seen in Figure 2.

**Figure 3:**
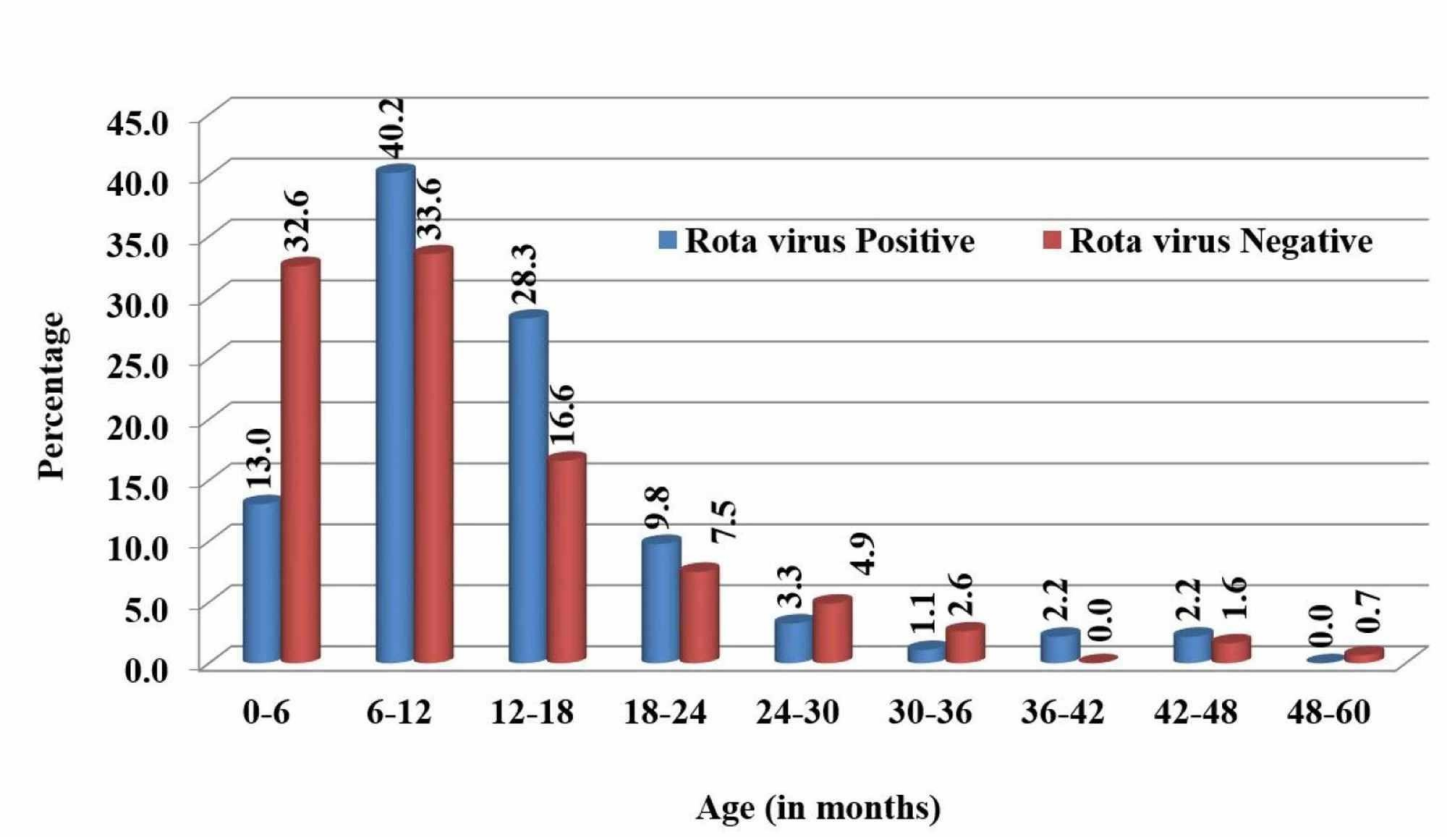
Age distribution of patients with rotavirus and non-rotavirus diarrhea

Of the 92 rotaviral diarrhea patients maximum number were found in 6-12 months age group 40.21% (n=37), followed by 12-18 months 28.26% (n=26), 0-6 months 13.04% (n=12), 18-24 months 9.78% (n=9), 24-30 months 3.26% (n=3), 36-42 months 2.17% (n=2), 42-48 months 2.17% (n=2), 30-36 months 1.08% (n=1), 48-60 months 0.00% (n=00). When compared with non rotaviral group, significant difference was found in age distribution of both the groups (Chi-square test (x2)=24.48, p-value=0.001), as seen in Figure 3. 

In the current study, we found males with a higher burden of diarrhea compared to females (1.6:1). Rotavirus positivity was higher in males (59/246=23.98%) compared to females (33/153=21.15%) in patients of acute gastroenteritis. However, the observed difference is statistically insignificant (p-value=0.663).

**Figure 4:**
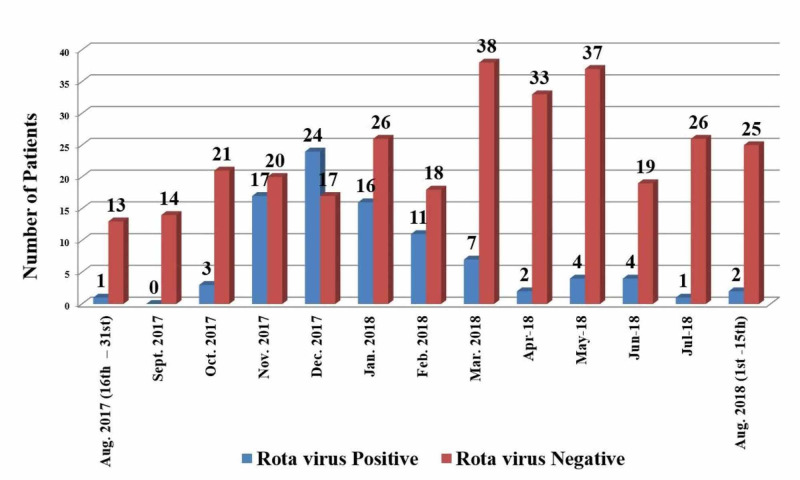
Month-wise distribution of diarrhea cases

Out of 92 rotavirus positive diarrhea cases, maximum cases were seen during winter months from November to February (n=68). The positivity was observed highest in December 2017 (26.08%) and lowest in September 2017 (00%) Chi-square test (x2)=77.98, p-value=0.0001, shows a significant association of rotavirus with colder months than with hotter months, as seen in Figure 4.

This study determined that 63.04% and 36.95% of rotavirus cases were from urban and rural areas, respectively, with statistically non-significance (p=0.236).

In this study, a higher incidence of rotavirus positive cases was found in those using tap to house (78.26%) than those used shared community taps (14.13%). It indicates there is a higher frequency of rotavirus infection in those using tap to house than shared community taps, which was statistically non-significant (Chi-square (x2)=4.398, df=4, p-value=0.354).

**Figure 5:**
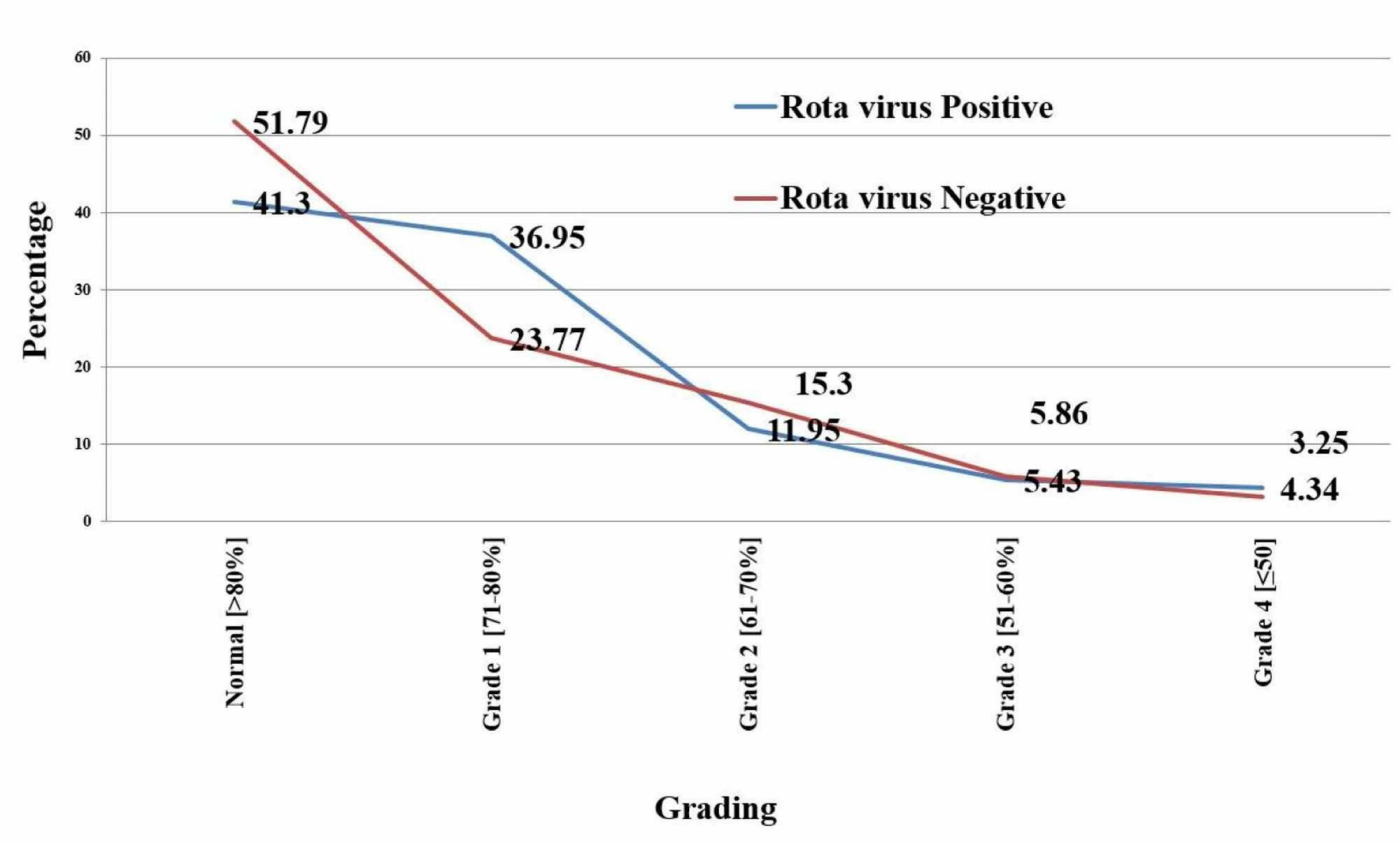
Distribution of cases according to the grade of malnutrition

In rotaviral diarrhea cases, grade 1 malnutrition was seen in 34 (36.95%) cases, grade 2 malnutrition in 11 (11.95%) cases, grade 3 malnutrition in 5 (5.43%) cases, and grade 4 malnutrition 4 (4.34%) cases. In the non-rotavirus group, 73 (23.77%) cases had grade 1 malnutrition, 47 (15.30%) had grade 2 malnutrition, 18 (5.86%) had grade 3 malnutrition, and 10 (3.25%) cases had grade 4 malnutrition, as seen in Figure 5.

Fever was present in 29 (31.52%) cases of rotavirus diarrhea and 126 cases (41.04%) of non-rotavirus diarrhea. When compared in both groups, the presence of fever was not statistically significant (p-value=0.128). Vomiting was present in 82.60% of positive rotavirus cases and 68.07% of negative rotavirus cases, which was statistically significant (p-value=0.01).

Duration of diarrhea in rotavirus cases was 1-2 days in 56 (60.86%) patients, 2-4 days in 30 (32.60%) patients, 4-6 days in 5 (5.43%) patients, and 6-7 days in 1 (1.08%) patient. In non-rotaviral group, duration was 1-2 days in 207 (67.42%), followed by 2-4 days 75 (24.42%), 4-6 days 25 (8.14%), and 6-7 days in 0 (0%) cases. There was a statistically non-significance difference in the diarrhea duration observed in both groups (X2=6.289, df=3, p=0.098).

In cases with rotaviral diarrhea, some dehydration was present in 67 (72.82%) cases; severe dehydration was present only in 4 (4.34%) cases and no dehydration in 21 (22.82%) cases. In cases with non-rotaviral diarrhea, some dehydration was seen in 247 (80.45%) cases, severe dehydration in 12 (3.90%), and no dehydration in 48 (15.63%) cases. The study found cases of rotavirus with some dehydration (X2=2.674, p=0.262, df=2).

Treatment of the patients in the study group was done as per WHO guidelines according to the degree of dehydration. Intravenous fluids were required in 79 (85.86%) patients with rotavirus diarrhea and 275 (89.57%) patients with non-rotavirus diarrhea. 32 (10.42%) patients with non-rotavirus diarrhea and 13 (14.13%) patients with rotavirus diarrhea did not require intravenous fluids (Odds Ratio (OR)=0.70, p=0.424), which were statistically not significant. Oral rehydration solution (ORS) was given to all the 92 patients. Oral zinc was given for 14 days in all the patients.

In this study, the maximum patient in both groups required hospitalization for 1-5 days. However, 16 and 5 patients in the non-rotavirus positive group required hospitalization for 10-15 days and 15-20 days, respectively, because of associated comorbid conditions. Due to this fact, a significant difference in duration of mean stay was observed (4.44±1.86 days vs. 5.21±3.03 days).

## Discussion

Overview

Acute watery diarrhea continues to be a challenge among infants and children as an important cause of mortality and morbidity. Our study observed that the prevalence of rotavirus diarrhea in children below five years of age is 23.05%. The result of this study supports other studies conducted by Bahl et al., which determined rotavirus in 23.5% of stool samples of children suffering from diarrhea in New Delhi [5]. Mathew et al. in Ernakulam district, Kerala, also detected rotavirus in 35.9% of diarrhea-related hospital admissions among children less than five years of age [6]. The study by Forster et al. in Europe detected the prevalence of rotavirus diarrhea 43.4% among children less than five years of age [7].

The most susceptible age group for the rotavirus infection was found to be of children less than 24 months. Our study determined that 37 (40.21%) of positive rotavirus cases were in the age group 6-12 months, followed by 35 (38.04%) cases in 13-24 months. Similar results were reported in a study by Mathew et al., who reported high prevalence in children aged 6 -11 months and 12-23 months (31.9% and 41.9%, respectively) [6].

In the present study, male and female children constituted 64.13 % (59) and 35.86% (33), respectively, of total children suffering from rotavirus diarrhea (92). It also found that males were infected higher compared to female children, with an estimated ratio of 1.79:1. According to other studies like Banerjee et al., a larger proportion of children admitted in the hospital due to rotavirus diarrhea was male (63.8%) and male to female ratio of 1.76:1 [8].

In this study, a higher number of rotavirus cases were observed in colder months than hotter months. There was a higher prevalence during November to February (68 out of 92). A similar trend was also confirmed in a study by Bahl et al., where rotavirus hospitalization incidence peaked in winter (i.e., from November through February) [5]. In contrast, Banerjee et al. reported no significant seasonal trends in their study done in Vellore [8]. However, they found a peak of rotavirus diarrhea from July to September in 2003, corresponding to the rainy season. 

Geographical distribution of rotavirus

The prevalence of rotavirus infection was found to be higher in urban than in rural areas; however, it is found in all areas. Several factors contribute to the transmission of the infection, including early breastfeeding cessation, daycare centers, playschools, water sports, and food formulas. 

The prevalence of rotavirus in rural areas is attributed to a lack of awareness among the rural population regarding hygiene and improper waste disposal, which are considered the significant risk factors for diarrheal diseases in children below five years. Also, insufficient hygiene measures, which include hand washing prior to food preparation and feeding their children, contribute to the higher prevalence rate. Furthermore, environmental issues related to the open defecation impact adversely in the control of rotavirus cases. 

Rotavirus infection and water supply

There was a higher frequency of rotavirus infection in those using tap to house water, shared community tap, boreholes, and covered well, consisting of 78.26%, 14.13%, 5.43%, and 2.17%, respectively. Maximum diarrhea cases and rotavirus positivity were seen in children who consumed tap water, which can be explained by whether this water was contaminated or had poor filtration. Drinking water from an unsafe source is one of the most important and common causes of rotavirus infections in developing countries [9-10]. The waterborne acute gastroenteritis due to Group B rotavirus is mainly found amongst children and adults of India, Bangladesh, Myanmar, and China [1,11]. 

Malnutrition

In our study, malnutrition was absent in 38 (41.30%) cases with rotavirus diarrhea and 159 (51.79%) patients with non-rotavirus diarrhea. The grade 1 malnutrition was present in 34 (36.95%) patients with rotaviral diarrhea and 73 (23.77%) in the non-rotaviral diarrhea group. Grade 2 malnutrition was found in 11 (11.95%) patients with rotavirus diarrhea and 47 (15.30%) patients with non-rotavirus diarrhea. The grade 3 malnutrition was observed in five (5.43%) cases with rotavirus diarrhea and 18 (5.86%) cases with non-rotavirus diarrhea. And grade 4 malnutrition found in four (4.34%) patients of rotavirus diarrhea and 10 (3.25%) patients of non-rotavirus diarrheal group. Studies observed no significant difference in the prevalence of rotavirus diarrhea and the severity of dehydration in children with or without malnutrition in Bangladesh [1,12].

Clinical features

We found that fever was present in 29 (31.52%) patients, vomiting in 76 (82.60%) patients, and diarrhea in all 92 (100%) patients with rotavirus gastroenteritis. The duration of diarrhea was for 1-6 days in 91 (98.91%) cases. Similar results were seen in the study conducted by Azemi et al., with the most dominant symptom diarrhea (98.59%), was followed by vomiting (88.02%). On average, diarrhea lasted for five days [13].

We observed that the degree of dehydration has a very significant correlation with the presence and absence of rotavirus diarrhea. Most of the rotavirus cases had some dehydration in 67 (72.82%) cases, and only 4 (4.34%) cases with rotavirus diarrhea had severe dehydration. Mathew et al. observed that moderate dehydration was present in 49.29% of cases, 14.08% had severe dehydration [6]. In 2010, a study in Nepal conducted by Sherchand et al. noted that the dehydration in rotavirus cases was likely to be moderate to severe than mild [14].

Rotavirus genotyping

In the current study, the results showed that G3P8 was the predominant strain (55.43%), followed by G1P8 (9.72%) and G3+G12P8 (8.69%), whereas the study conducted by Mathew et al. who reported that G1P8 (49.7%) was the most common strain identified followed by G9P8 (26.4%), G2P4 (5.5%), G9P4 (2.6%) and G12P6 (1.3%) [6]. Another study conducted by Forster et al. showed that the most common strains were G1P8 (40.3%), followed by G9P8 (31.2%), G4P8 (13.5%), G3P8 (7.1%) [7].

Another study identified a rare emerging strain of G11P25 [15]. From Pune, G9P8, G12P6, and G12P8 were isolated in another study [16]. The research reported that G2P4 was predominant than G1P8 in 2007, and G12P6, as well as G12P8, emerged only during that period [17]. The difference in identified strain in our study compared to others highlights the genetic diversity of rotavirus.

Treatment and management

Treatment was determined based on the degree of dehydration and the clinical condition of the patients. Intravenous fluids were needed in 79(85.89%) patients in rotavirus diarrhea and 275 (89.57%) non-rotaviral diarrhea cases. Oral rehydration therapy (ORT) was given in 100% (92) cases of Rotavirus diarrhea. Oral zinc was given for 14 days in all the patients. Excessive use of IV fluids in our study in case of some dehydration was due to parental concern, which was given for the initial few hours of admission in most of the cases followed by ORT for the remaining hospitalization period. Dehydration of 5% to 10% due to viral or bacterial diarrhea can be managed effectively and safely through oral rehydration [1,18].

Our study determined the mean hospital stay in rotavirus diarrhea was 4.44±1.86 days compared to 5.21±3.03 days in the non-rotaviral group. A study by Kurugol et al., on the other hand, observed that the mean hospital stay for rotavirus gastroenteritis was significantly longer, 5.5±5.1 days compared to 3±3.1 days for non-rotavirus gastroenteritis [19]. Our result was the reverse of the above study, which can be explained by associated comorbid conditions requiring prolonged hospitalization in non-rotaviral groups.

Recommendations

Rapid diagnosis through identifying the Rota antigen provides crucial evidence of the infection to the clinicians. Early diagnosis may also prevent inappropriate administration of antibiotics and help prevent infection spread, particularly in institutions. Detecting the rotavirus genotype assists in epidemiological studies and surveillance. Identifying the molecular level of rotavirus infection also helps to limit the spread of infection by preparing a potent vaccine based on the prevalent strain.

## Conclusions

We concluded that rotavirus diarrhea accounts for the major cause of mortality and morbidity in children in the age group of six months to two years, especially in developing countries. Rotavirus diarrhea is significantly prevalent in 92 (23.05%) cases in our study. The maximum number of cases are seen from November to February. A significant number of rotaviral diarrhea have some dehydration. On the molecular characterization of positive rotavirus samples, G3P8 (55.43%) followed by G1P8 (9.72%) and G3G12P8 (8.69%) were the most common serotypes isolated in the study. We determined no statistically significant difference comparing the occurrence of diarrhea and its severity amongst children who are vaccinated and non-vaccinated.
